# A Simple Schiff Base Probe for Quintuplicate-Metal Analytes with Four Emission-Wavelength Responses

**DOI:** 10.3390/molecules28176400

**Published:** 2023-09-01

**Authors:** Jingzhe Zhang, Kaili Wang, Yilu Sun

**Affiliations:** 1 Key Laboratory of Environmental Biotechnology, Research Center for Eco-Environmental Sciences, Chinese Academy of Sciences, Beijing 100085, China; zjz0307@163.com; 2 Beijing Municipal Research Institute of Eco-Environmental Protection, Beijing 100037, China; 3 University of Chinese Academy of Sciences, Beijing 100049, China; 4 State Environmental Protection Engineering (Beijing) Center for Industrial Wastewater Pollution Control, Beijing 100037, China

**Keywords:** single molecular probe, four-emission, quintuplicate-analytes, logic gate

## Abstract

A versatile mono-Schiff compound consisting of *o*-aminobenzene-hydroxyjulolidine (ABJ-MS) has been easily synthesized using a one-step reaction. ABJ-MS displays four diverse fluorescence responses to the addition of Zn^2+^/Al^3+^/Fe^3+^/Ag^+^, with the maximum fluorescence emission at 530 nm undergoing a hypsochromic shift to 502/490/440/430 nm, synchronously with the discriminating fluorescence enhancement being 10.6/22.8/2.6/7.1-fold, respectively. However, the addition of Cu^2+^ into ABJ-MS leads to an opposite behavior, namely, fluorescence quenching. Meanwhile, ABJ-MS also displays distinct absorption changes after adding these five metal ions due to different binding affinities between them and ABJ-MS, which gives ABJ-MS quite a versatile detecting nature for Cu^2+^/Zn^2+^/Al^3+^/Fe^3+^/Ag^+^. Moreover, ABJ-MS can mimic a series of versatile AND/OR/INH-consisting logic circuits on the basis of the Cu^2+^/Zn^2+^/Al^3+^/Fe^3+^/Ag^+^-mediated diverse optical responses. These will endow the smart ABJ-MS molecule and potential applications in the multi-analysis chemosensory and molecular logic material fields.

## 1. Introduction

As is well known, metal ions play a quite important role in diverse physiology and industry processes, although they could also cause some serious diseases in excessive/deficient conditions [1,2]. For example, Zn^2+^ as an essential element has taken part in some physiological activities. Yet, the insufficiency/surfeit of zinc ions could cause Friedreich’s ataxia, Alzheimer’s and Parkinson’s diseases [3,4]. Similarly, excessive/deficient Cu^2+^ could bring about certain neurological sickness and gastrointestinal disturbances, Fe^3+^ may induce liver and kidney diseases as well as metabolism maladjustment, while Al^3+^ can cause some damage to human′s central nervous and immune systems [5,6,7,8]. Ag^+^ accumulation could also cause serious consequences, such as grayish-blue skin discolorations [9]. Furthermore, excessive metal ions in domestic/industrial wastewater could induce bad environmental pollution. Thus, developing highly effective methods to detect these metal ions is extraordinarily necessary.

Among numerous analytic techniques, fluorescent probes, particularly those with detecting functions for some important metal ions, are being paid growing attention to due to their rapid responsiveness, on-site real monitoring, and high sensitivity [10,11,12]. However, most fluorescent probes concentrate on one-to-one detections, while only a few studies have focused on one-to-multi-sensing, either simultaneously or separately [13,14,15,16,17]. Nevertheless, the single molecular probes for multi-analytes could not only shorten the preparation procedure for multiple probes but also exhibit a high detecting efficiency compared to single-analyte detecting sensors [18,19,20,21]. Thus, it is extremely desirable to explore and develop single fluorescent probes for multiple targets through diverse optical responses. To actualize the versatile monomolecular detection for multiple targets, generally, more than one luminophore or receptor is combined into the single-molecular probe through a laborious multistep organic synthetic procedure [22,23]. Suzuki and co-workers prepared a probe consisting of tris-fluorophores to detect Fe^3+^/Pb^2+^/Al^3+^/Cu^2+^ based on the diverse complexing modes between receptors and metal ions [24]. Akkaya et al. synthesized a Bodipy probe for Zn^2+^/Ca^2+^/Hg^2+^, consisting of three receptors [25], while the Jiang group prepared a one-receptor-bridging porphyrin-Bodipy dyad probe for Fe^2+^/Hg^2+^ [26]. Meanwhile, few investigations have been conducted on probes consisting of single-fluorophore-receptors for multi-analytes associated with diverse detecting mechanisms, etc. [27,28,29,30]. However, to the best of our knowledge, there still appears to be no exploration into a molecular probe with only one luminophore and a single receptor, except for quintuplicate-metal ions, especially with the easy-to-synthesize process.

**Scheme 1:**
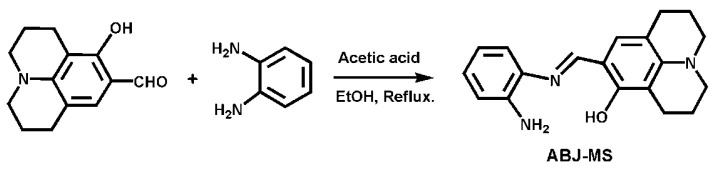
Synthesis of ABJ-MS.

Meanwhile, ortho-hydroxy keto–imines are receiving increasing attention owing to their wide application [31,32]. Among them, the salicylaldazine moiety has been one of the most promising construction units for fluorescent probes due to its excellent metal complexing affinity, its unique enol-keto tautomerization, and C=N isomerization, which could induce sensitive optical changes [33,34,35]. In addition, the metal-binding affinity and optical-signal sensibility can be easily improved by the introduction of a third binding site onto the salicylaldazine moiety [36,37]. Herein, we designed and synthesized a new three-in-receptor mono-Shiff molecule (ABJ-MS) by introducing an *o*-aminobenzene unit onto the salicylaldazine moiety based on the mono-condensation reaction between *o*-diaminobenzene and 9-formyl-8-hydroxyjulolidine, Scheme 1. As expected, ABJ-MS displays five discriminating fluorescence behaviors with four emission wavelengths after adding Zn^2+^, Cu^2+^, Al^3+^, Fe^3+^, or Ag^+^, which are simultaneously accompanied by the significantly different absorption responses, owing to different binding affinities between these five metal ions and ABJ-MS. In addition, a series of complicated logic circuits consisting of AND/OR/INH gates could be mimicked for the ABJ-MS on the basis of the Cu^2+^/Zn^2+^/Al^3+^/Fe^3+^/Ag^+^-mediated diverse optical responses. These will endow the ABJ-MS molecule and the potential applications in the versatile multi-analysis chemoprobe and the molecular logic material fields.

## 2. Results

The aimed mono-Schiff probe consisting of *o*-aminobenzene-hydroxyjulolidine and a three-in-receptor (ABJ-MS) (Scheme 1) was obtained by the mono-condensation of the *o*-diaminobenzene with the 8-hydroxyjulolidine-9-carboxaldehyde (1:1 equiv.), in ethanol and under a refluxing condition. The ABJ-MS compound was characterized by NMR, elementary analysis, and mass spectroscopies, as shown in Figures S1 and S2 (Supporting Information).

### 2.1. The Selectivity Ability

To understand the optical properties of ABJ-MS, the electronic absorption and fluorescence emission behaviors of this compound (20 μM) in a DMSO–H_2_O mixture with different H_2_O fractions (*f*_w_, the volume percentage of H_2_O in DMSO-H_2_O mixtures) were studied. As shown in Figure S3 (Supporting Information), when the water fraction *f*_w_ was below 60%, the electronic absorption of ABJ-MS undergoes minimal changes. Whereas, when *f*_w_ was further increased from 60 to 95%, the maximum absorption of ABJ-MS becomes slightly broader and red-shifted, and in the meantime, the level-off tail gradually appears in the long-wavelength region, accompanied by a slight decrease in the absorbance, which is possibly due to the Mie light scattering, and suggests the formation of aggregated nanoparticles. Meanwhile, along with the increase in *f*_w_ from 0–60% to 60–5%, the weak fluorescence emission of ABJ-MS was observed to be firstly enhanced and then decreased, probably due to the formation of aggregated nanoparticles, which inhibit the intramolecular rotation motion and enhance the intermolecular π–π stacking interaction, thereby resulting in aggregation-caused quenching in the high *f*_w_ conditions [38]. Thus, the sensing properties of ABJ-MS were investigated in a mixture of DMSO/H_2_O (*v*:*v* = 1:4).

**Figure 1:**
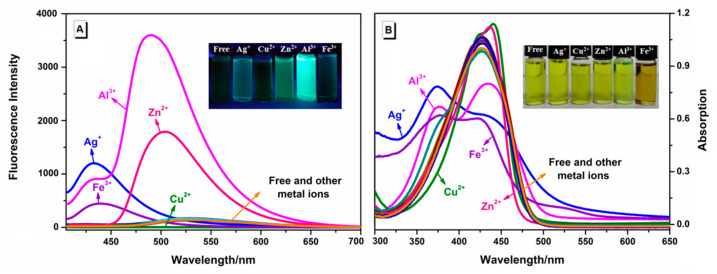
(**A**) Fluorescence emission spectra and (**B**) electronic absorption spectra of ABJ-MS (20 µM) upon addition of different metal ions, such as Hg^2+^, Pb^2+^, Co^2+^, Mn^2+^, Ni^2+^, Cd^2+^, Ca^2+^, Ba^2+^, Mg^2+^, Li^+^, Na^+^, K^+^, Ag^+^ (blue), Zn^2+^ (pink), Cu^2+^ (green), Al^3+^ (magenta), or Fe^3+^ (violet) (10 equiv.) in mixed DMSO/H_2_O (4:1).

To identify the detecting capability of this compound for metal ions, the optical properties of ABJ-MS (20 μM) were investigated upon the addition of metal ions, such as Zn^2+^, Cu^2+^, Al^3+^, Fe^3+^, Ag^+^, Hg^2+^, Pb^2+^, Co^2+^, Mn^2+^, Ni^2+^, Cd^2+^, Ca^2+^, Ba^2+^, Mg^2+^, Li^+^, Na^+^, or K^+^ (10 equiv.), in DMSO/H_2_O (4:1). As can be found from Figure 1, the absorption and fluorescent spectra of ABJ-MS remained almost unchanged after adding the above metal ions, except for Zn^2+^, Al^3+^, Fe^3+^, Ag^+^, or Cu^2+^. After adding Zn^2+^/Al^3+^/Fe^3+^/Ag^+^, the weak fluorescence emission of ABJ-MS displayed diverse responses: The maximum emission wavelength of ABJ-MS (530 nm) underwent a hypsochromica shift to 502/490/440/430 nm, which was synchronously accompanied by a discriminating 10.6/22.8/2.6/7.1-fold increase in fluorescence emission, respectively, alongside an increase in the relative fluorescence quantum yield from 0.005 of free ABJ-MS to 0.040, 0.107, 0.010, and 0.024 upon the addition of Zn^2+^, Al^3+^, Fe^3+^, and Ag+, respectively [39]. On the contrary, the addition of Cu^2+^ induces the fluorescence quenching, Figure 1A. Meanwhile, the addition of these five metal ions into ABJ-MS also causes obviously different changes in absorption spectra. After adding Zn^2+^ ions, the absorption at 427 nm from ABJ-MS undergoes a bathochromic shift to 437 nm following a slight enhancement in absorbance. This is also true for the addition of Cu^2+^, although the maximum absorption appears at 441 nm. Differently, after adding Al^3+^, the absorption of ABJ-MS at 427 nm decreased simultaneously with a bathochromic shift to 436 nm as well as a slightly weak shoulder band appearing around 376 nm. The addition of Ag^+^ also induced a similar change but with the maximum absorption appearing at 373 nm, thereby resulting in the varying ratio of *A*_427_/*A*_373_ from 2.8 to 0.7. After adding Fe^3+^, the above-mentioned maximum absorption for ABJ-MS decreased simultaneously alongside an obvious increase at 377 nm, which led to a broad absorption in the range of 310–490 nm, Figure 1B. These results endow ABJ-MS as a quite versatile detecting nature for Zn^2+^/Cu^2+^/Al^3+^/Fe^3+^/Ag^+^ that is associated with five discriminating fluorescence and absorption dual-responses, which is quite rare in reported fluorescence probes.

### 2.2. The Responsive Metal Sensing

**Figure 2:**
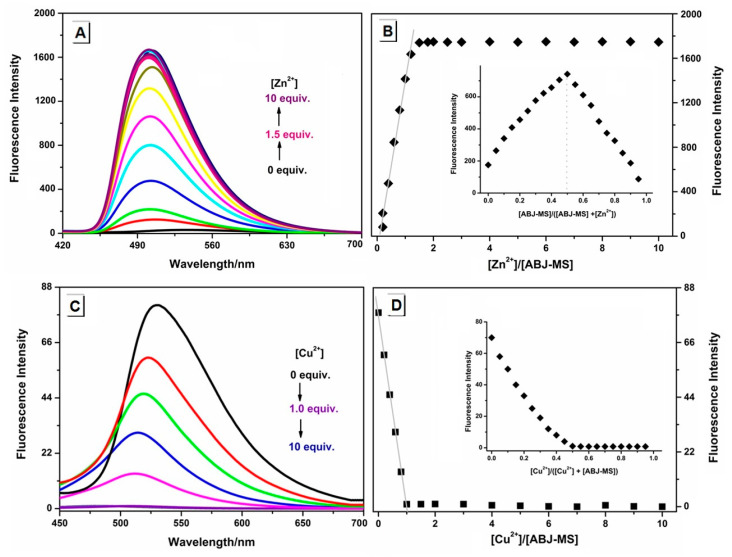
The fluorescence emission and intensity changes for ABJ-MS with increasing Zn^2+^ (**A**,**B**) and Cu^2+^ amounts (**C**,**D**) (0 to 10 equiv.); the insets are fluorescent Job’s plots of ABJ-MS in nm, showing 1:1 binding stoichiometry between ABJ-MS and Zn^2+^ (**B**) or Cu^2+^ (**D**).

Zn^2+^/Cu^2+^ sensing: The fluorescent titration experiment for ABJ-MS (20 µM) was carried out by increasing the Zn^2+^ content in the DMSO/H_2_O (4:1) solution. As can be seen in Figure 2A, along with the increase of Zn^2+^ quantity from 0 to 1.5 equiv., the fluorescence emission from ABJ-MS gets gradually increased with a hypsochromic shift from 530 nm to 502 nm, whereas the strong absorption by this compound is found to undergo a bathochromic shift to 437 nm. Subsequently, the fluorescent emission and absorption spectra remain almost unchanged upon the continuous increase in Zn^2+^ to 10 equiv., Figure S4 (Supporting Information). Furthermore, the fluorescence emission (*F* − *F_min_*) of ABJ-MS raised linearly along with the increase in Zn^2+^ from 0 to 1.5 equiv., on the basis of the Benesi–Hildebrand equation, Figure 2B, suggesting a probable 1:1 complexing between ABJ-MS and Zn^2+^, where the approximate association constant (*K_a_*) was 3.04 × 10^4^ [40], which is further affirmed in the fluorescent Job’s plot, Figure 2B. In addition, the ESI–mass spectrometry for ABJ-MS upon the addition of Zn^2+^ (10 equiv.) produces a peak at *m*/*z* = 388.42, which is assignable to [ABJ-MS + Zn^2+^ + H_2_O + H]^+^ (calculated at 388.1), Figure S5 (Supporting Information); thus, again confirming the 1:1 complexing between ABJ-MS and Zn^2+^. Notably, despite the fluorescence quenching response following the addition of Cu^2+^, the fluorescent titration experiment of ABJ-MS (20 µM) with Cu^2+^ (0–10 equiv.) also displays a similar 1:1 binding stoichiometry for the ABJ-MS-Cu^2+^ system, yet with an approximate *K_a_* of 1.2 × 10^5^, Figure 2C,D. The detection limits of ABJ-MS for Zn^2+^ and Cu^2+^ ions were determined as 3.21 × 10^−^^7^ and 1.06 × 10^−^^7^ M under the present condition, which was lower than those applied to the potable water by the WHO [41], suggesting that ABJ-MS could be a highly sensitive probe for Zn^2+^ and Cu^2+^ using the diverse fluorescence off–on and on–off signals, respectively.

**Figure 3:**
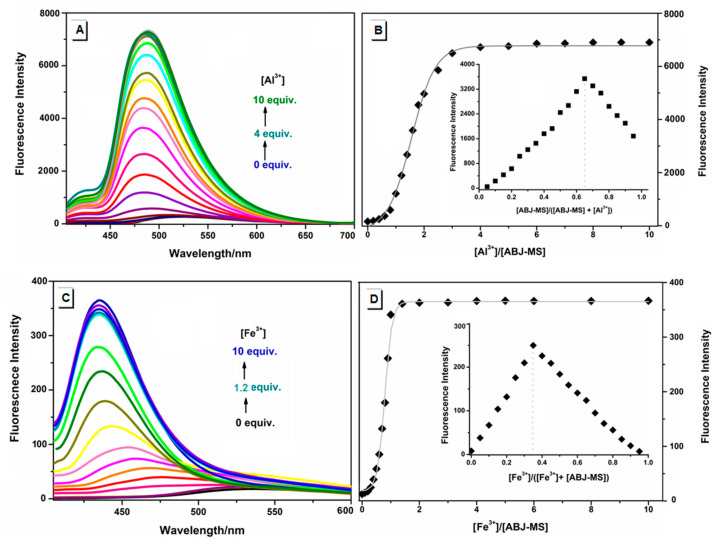
The fluorescence emission and intensity changes for ABJ-MS along with the increasing amounts of Al^3+^ (**A**,**B**) and Fe^3+^ (**C**,**D**), from 0 to 10 equiv.; the insets are fluorescent Job’s plots of ABJ-MS with Al^3+^ and Fe^3+^ (490 and 440 nm), showing 2:1 binding stoichiometry between ABJ-MS and Al^3+^/Fe^3+^, respectively.

Fe^3+^/Al^3+^ sensing: As shown in Figure 3A, the fluorescent titration test for ABJ-MS with Al^3+^ was also studied by successively increasing Al^3+^ (0–10 equiv.) in the DMSO/H_2_O mixture (4:1). Alongside increasing the Al^3+^ amount from 0 to 4 equiv., the weak fluorescence emission from ABJ-MS gets gradually increased with a synchronous hypsochromic shift from 530 to 490 nm, resulting in a fluorescence intensity ratio of 25:1 for *F*_490_*/F*_530_. Meanwhile, the changes in the absorption for ABJ-MS mainly focus on the added Al^3+^ amount being 0–4 equiv., Figure S6 (Supporting Information). Furthermore, the maximum intensity in the fluorescent Job’s plot for ABJ-MS with Al^3+^ was observed when the molar fraction of (ABJ-MS) vs. (Al^3+^) + (ABJ-MS) was 0.66, suggesting the possible 2:1 complexing of ABJ-MS with Al^3+^, Figure 3B [42]. Based on the 2:1 binding stoichiometry and fluorescence titration experiments of ABJ-MS with Al^3+^, the binding constant for this compound with Al^3+^ was estimated to be 9.52 × 10^3^, by plotting the Benesi–Hildebrand equation lg[(*F_l_* − *F*_0_)/(*F* − *F*_0_)] against lg(1/(Al^3+^)) [43,44]. Similarly, the fluorescence titration experiment and fluorescent Job’s plot for ABJ-MS with Fe^3+^ also showed a similar 2:1 binding mode between ABJ-MS and Fe^3+^, with an approximate *K_a_* of 8.72 × 10^4^, Figure 3C,D. The detection limits of ABJ-MS for Al^3+^ and Fe^3+^ ions were determined as 9.07 × 10^−^^7^ and 2.69 × 10^−^^7^ M, respectively, indicating the sensitively diverse fluorescence off–on sensing function of ABJ-MS to Al^3+^/Fe^3+^.

**Figure 4:**
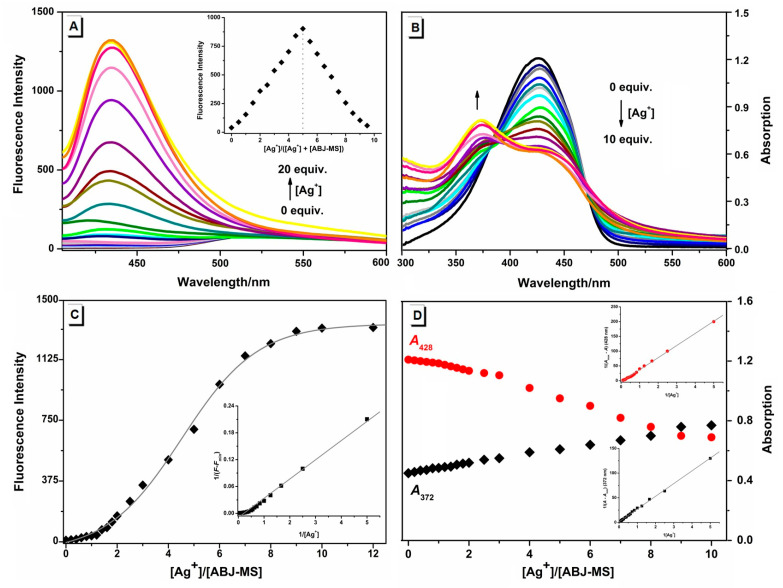
(**A**) The fluorescence emission spectra and (**B**) absorption spectra of ABJ-MS alongside the increasing Ag^+^ amount (0–10 (or 12) equiv.); the fluorescence intensity changes and linear increase of 1/(*F* − *F*_min_) against 1/(Ag^+^) at 430 nm (**C**); the absorbance changes and linear increase of 1/(*A* − *A*_min_) or 1/(*A_max_* − *A*) against 1/(Ag^+^) at 373 and 427 nm (**D**), respectively. The inset is a fluorescent Job’s plot of ABJ-MS with (Ag^+^) showing 1:1 binding stoichiometry between ABJ-MS and (Ag^+^) in (**A**).

Ag^+^ sensing: As shown in Figure 4A, the fluorescence emission by ABJ-MS at 430 nm gradually increases as the Ag^+^ content increases from 0 to 10 equiv., and the corresponding 1/(*F* − *F_min_*) raises linearly against the alteration in 1/(Ag^+^) (0–12 equiv.), according to the Benesi–Hildebrand equation, Figure 4B. Moreover, the absorption titration for ABJ-MS with Ag^+^ shows that both absorptions 1/(*A* − *A_min_*) and 1/(*A_max_* − *A*) at 373 and 427 nm, respectively, by ABJ-MS also increased linearly against the change of 1/(Ag^+^) (0–10 equiv.), with an approximate *K_a_* of 1.6 × 10^3^ M^−1^, Figure 4C,D [45]. These results indicate a possible 1:1 stoichiometry between ABJ-MS and Ag^+^, which is again approved by the fluorescent Job’s plot of ABJ-MS towards Ag^+^, Figure 4A. The detecting limitation by ABJ-MS for Ag^+^ is 1.03 × 10^−^^5^ M, suggesting an excellent detecting function for ABJ-MS towards Ag^+^ through the fluorescence off–on and absorption dual-channels.

### 2.3. Competition Experiment

**Figure 5:**
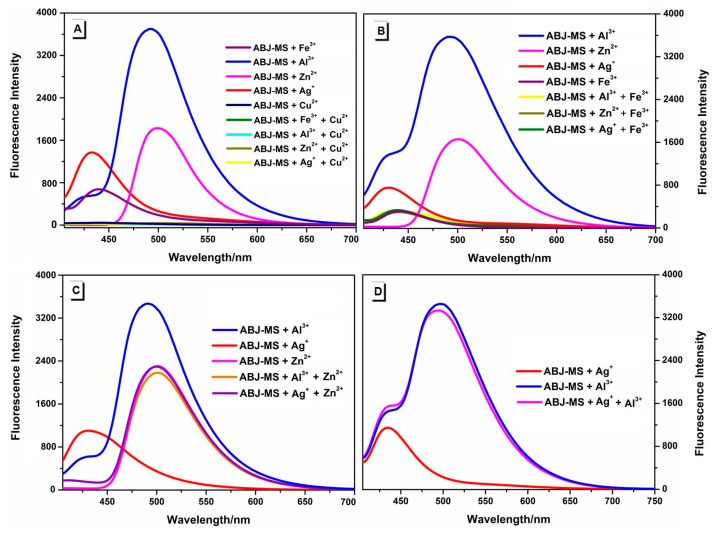
The fluorescence emission spectra of ABJ-MS–Fe^3+^/Zn^2+^/Al^3+^/Ag^+^ complexes (1:10) upon addition of Cu^2+^ (**A**); ABJ-MS–Zn^2+^/Al^3+^/Ag^+^ complex (1:10) upon addition of Fe^3+^ (**B**); ABJ-MS–Al^3+^/Ag^+^ complex (1:10) upon addition of Zn^2+^ (**C**); ABJ-MS–Ag^+^ complex (1:10) upon addition of Al^3+^ (**D**).

The competition experiments were carried out in DMSO/H_2_O (4:1) mixtures to further study the detecting abilities for ABJ-MS of Zn^2+^/Cu^2+^/Al^3+^/Fe^3+^/Ag^+^. As can be observed in Figure 5A and Figure S7 (Supporting Information), the fluorescent emission of the ABJ-MS-Fe^3+^ (1:10) complex at 440 nm is quenched after adding Cu^2+^ (10 equiv.); meanwhile, the absorption spectrum is observed to be similar to the ABJ-MS-Cu^2+^ complex, suggesting the substitution of Fe^3+^ by Cu^2+^ in the ABJ-MS–Fe^3+^ composite, which is also true for the ABJ-MS–Zn^2+^/Al^3+^/Ag^+^ complexes. Similarly, the addition of Fe^3+^ into the ABJ-MS–Zn^2+^/Al^3+^/Ag^+^ systems also results in the displacement of metal in these systems by Fe^3+^ as well as the consequential fluorescence response, Figure 5B. In a similar manner, Zn^2+^ can replace the metal ion in the ABJ-MS–Al^3+^/Ag^+^ complex and Al^3+^ can replace the metal ion in the ABJ-MS–Ag^+^ complex, thereby inducing the corresponding fluorescence and absorption responses, Figure 5C,D, Figures S8 and S9 (Supporting Information). By contrast, after adding other metal ions, the fluorescence emission of the ABJ-MS–Cu^2+^/Fe^3+^/Zn^2+^/Al^3+^/Ag^+^ systems remained almost unchanged, Figures S10–S14 (Supporting Information). These results show the excellent binding affinity between ABJ-MS and these five metal ions, in the order of Cu^2+^ > Fe^3+^ > Zn^2+^ > Al^3+^ > Ag^+^, which is in good agreement with their association constant results mentioned, thereby revealing the favorable selectivity of ABJ-MS for Cu^2+^/Fe^3+^/Zn^2+^/Al^3+^/Ag^+^ over other tested metal ions. In addition, the selectivity of ABJ-MS toward anions was also investigated through the addition of sodium salts and different anions, including F^−^, Cl^−^, Br^−^, I^−^, HSO_3_^−^, OAc^−^, S^−2^, SO_3_^−2^, SO_4_^−2^, CO_3_^−2^, HPO_4_^−2^, and H_2_PO_4_^−^. Notably, the electronic absorption and fluorescence emission spectra of ABJ-MS made either a slight or no change upon the addition of 100 equiv. of each anion, meaning little effect was shown by these anions on the sensing properties of ABJ-MS, Figure S15. Thus, ABJ-MS could be utilized as a versatile molecular probe for Cu^2+^/Fe^3+^/Zn^2+^/Al^3+^/Ag^+^ through dual-mode optical signals associated with immediate interactions between these five metal ions and ABJ-MS as well as the diverse metal-displacement of the ABJ-MS–M(Ag^+^/Al^3+^/Zn^2+^/Fe^3+^) complexes by Cu^2+^/Fe^3+^/Zn^2+^/Al^3+^, representing the quite rare example of smart single molecular probe for quintuple-metal analysis using the sensitive fluorescence and absorption dual-signals.

### 2.4. The Sensing Mechanism

As is well known, there is usually an equilibrium between the enol–imine and keto–enamine isomers for salicylaldazine moiety in solution due to the enol–keto isomerization, while the equilibrium moves easily under trace amounts of acid catalysis [46,47]. To understand the sensing mechanism between ABJ-MS and the corresponding metal ions, the optical behaviors of ABJ-MS after sequentially adding HCl were investigated under the same measuring requirement as mentioned above. As can be found from Figure S16 (Supporting Information), the increase in HCl content from 0 to 1 equiv., caused a bathochromic shift in the strong absorption of ABJ-MS from 427 to 438 nm with a little decrease in the absorbance. Then, continuously increasing the HCl amount, even to 100 equiv., caused the maximum absorption to decrease with a distinct enhancement in the absorption band at 368 nm. At the same time, the fluorescent emission for ABJ-MS at 530 nm was simultaneously decreased alongside the appearance of a new strong emission band at 434 nm along with the increasing HCl amount. The optical responses for ABJ-MS to the addition of HCl can be rationalized as follows: ABJ-MS may coexist as a mixture of enol–imine and keto–enamine isomers, although favors the keto–enamine isomer under a neutral environment, which is approved by the proton band at 13.52 ppm and is associated with an intramolecular hydrogen band (O…H-N) in the ^1^H NMR for ABJ-MS [4,30]. Upon the addition of HCl, the H^+^ firstly coordinates with the oxygen atom in the C=O unit from the keto–enamine isomer, which inhibits the intramolecular H-bonding interaction, promotes the conversion from the keto–enamine isomer to the enol–imine isomer, and results in a bathochromic shift in the maximum absorption from ABJ-MS [48,49]. Then, the nitrogen atom from the imine unit is protonated along with the increasing HCl amount, and the photoinduced electron transition is restrained, which in turn induces fluorescence enhancement by ABJ-MS.

**Figure 6:**
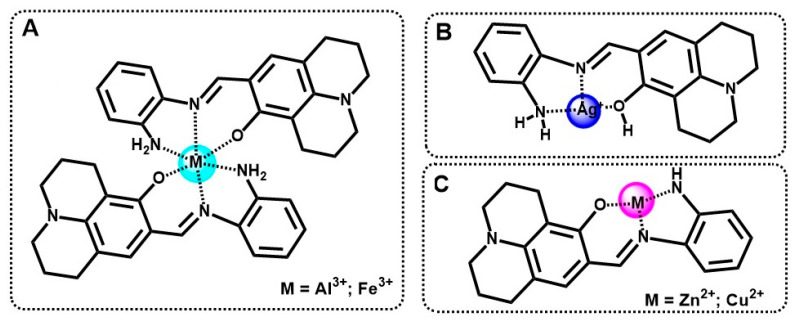
Subfigure (**A**) proposed coordination modes between ABJ-MS and Al^3+^or Fe^3+^; Subfigure (**B**) proposed coordination modes between ABJ-MS and Ag^+^; Subfigure (**C**) proposed coordination modes between ABJ-MS and Cu^2+^or Zn^2+^, with the other coordinated solution molecule or anion being omitted.

Notably, the addition of Al^3+^/Fe^3+^ into ABJ-MS has similar optical responses to those for ABJ-MS under the same acid conditions mentioned above. The responsive optical changes in the ABJ-MS–Al^3+^/Fe^3+^ system may be attributed to the hydrolysis of Al^3+^/Fe^3+^, which results in an acidic environment, promotes the equilibrium shift to the enol–imine form, and increases the complexing of ABJ-MS with Al^3+^/Fe^3+^. These changes, in combination with the 1:2 stoichiometry for the ABJ-MS–Al^3+^/Fe^3+^ complex, imply a possible binding mode between ABJ-MS and Al^3+^/Fe^3+^ ions, Figure 6A [50,51]. For the ABJ-MS–Ag^+^ complex, the quite similar optical responses to the ABJ-MS–HCl system, together with the 1:1 complexing, suggest a possible coordination mode between ABJ-MS and the Ag^+^ ions, Figure 6B. By contrast, upon the addition of Zn^2+^/Cu^2+^, there exist diverse changes in the absorption spectra of ABJ-MS, despite the similar red-shift in the maximum absorption, which could be attributed to the different binding mode for the ABJ-MS–Zn^2+^/Cu^2+^ complex, Figure 6C. The increase in fluorescence by ABJ-MS upon the addition of Zn^2+^/Al^3+^/Fe^3+^/Ag^+^ could be attributed to the restraint of both the C=N isomerization and the excited-state proton transfer (ESPT), in addition to the photoinduced electron transition process [52,53]. However, possibly due to the different characteristic outermost electronic structures between these responsive metal ions, the binding of ABJ-MS to Zn^2+^/Al^3+^/Fe^3+^/Ag^+^ induces a diverse fluorescence emission, whereas its coordination with Cu^2+^ results in fluorescent quenching, which is probably associated with its usual paramagnetic fluorescence quenching property, meaning that the ABJ-MS is quite a versatile multi-responsive molecular probe for Cu^2+^/Fe^3+^/Zn^2+^/Al^3+^/Ag^+^.

### 2.5. Application of ABJ-MS in Zn^2+^/Al^3+^/Fe^3+^ Analysis in Water Samples

Two kinds of water samples, such as from a river (Yongding River) and artificial water, were employed to explore the practical application potential of ABJ-MS. After performing a simple filtrate, there were no Zn^2+^/Al^3+^/Fe^3+^ according to the atomic absorption spectrometry. The water samples were spiked with a Zn^2+^/Al^3+^/Fe^3+^ solution and analyzed using the ABJ-MS molecular probe. As can be seen from Table 1, the results indicated the satisfying recovery and R.S.D. values for the tested samples using this newly prepared probe [54], endowing ABJ-MS with an improved potential application in sensing Zn^2+^, Al^3+^, and Fe^3+^.

### 2.6. The Construction of the Logic Gate

**Figure 7:**
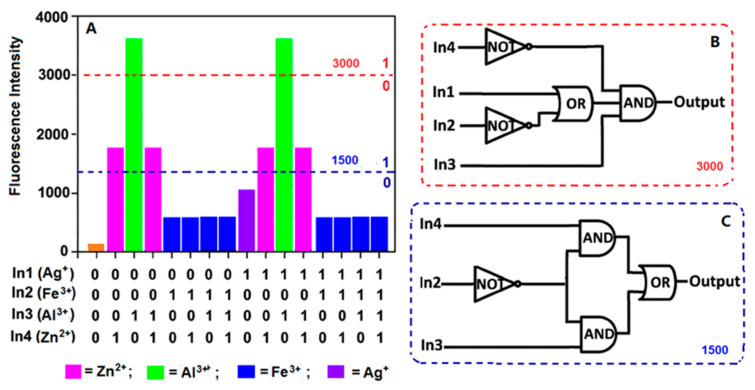
The truth value for ABJ-MS (20 µM) logic gate (**A**) and two complicated logic circuits (**B**,**C**) with 10 equiv. of Ag^+^, Fe^3+^, Al^3+^, and Zn^2+^ as four inputs (In1, In2, In3, and In4), respectively, the corresponding maximum fluorescence emission intensity as output, and the threshold level of fluorescence intensity being 3000 and 1500. Dotted lines represent the threshold levels for fluorescence outputs.

Inspired by the Cu^2+^/Fe^3+^/Zn^2+^/Al^3+^/Ag^+^ induction of diverse absorption and fluorescence changes, the smart ABJ-MS molecule was employed to construct logic systems. In whole logical operations, the ABJ-MS serviced as a gate, while Zn^2+^/Al^3+^/Fe^3+^/Ag^+^ were utilized as inputs, with the presence and absence being defined as 1 and 0, and the relevant maximum fluorescence emission intensity defined as outputs. Interestingly, a series of different function logic systems could be fabricated for ABJ-MS (20 µM) by rationally defining the logic threshold on the basis of the multiple stimulate-fluorescence signals. For example, when 10 equiv. of Zn^2+^ and Al^3+^ were utilized as inputs (In1 and In2), while the maximum fluorescence emission intensity (*F*max) for ABJ-MS upon the addition of metal ions was defined as outputs, then, two diverse logic gates will be fabricated with different threshold [55,56]: With *F*max at 3000 being the threshold to define the “1” (˃3000) and “0” (˂3000) states, the value of *F*max of ABJ-MS (output) becomes high (*F*_max_ = 3580, 1), yet only when the 10 equivalent of Al^3+^ (In2) was added. Conversely, it is at a low level (*F*_max_ ˂ 3000, 0) after the addition of Zn^2+^ (In1) as well as a combination of Al^3+^ and Zn^2+^; thus, mimicking an INHIBIT logic gate. Similarly, an OR logic gate was simulated using *F*_max_ at 1500 as the threshold, Figure S17 (Supporting Information). Notably, when the four metal ions Ag^+^, Fe^3+^, Al^3+^, and Zn^2+^ were utilized as inputs (In1, In2, In3, and In2) in combination with the two thresholds mentioned above, two complicated logic circuits consisting of AND–OR–NOT and OR–AND–NOT gates were mimicked according to the truth table, Figure 7 and Tables S1 and S2 (Supporting Information). Furthermore, using different Cu^2+^/Zn^2+^/Al^3+^/Fe^3+^/Ag^+^ combinations as inputs and diverse optical responses as outputs, a series of more complicated logic functions for ABJ-MS would be simulated by a rationally designed different threshold. Thus, the smart ABJ-MS fluorescent molecule not only exhibits the quite versatile detecting nature for Cu^2+^/Zn^2+^/Al^3+^/Fe^3+^/Ag^+^ but also could fabricate a series of complicated logic circuits, which endows the small ABJ-MS molecule with a wide potential of applications in the multi-function chemosensory and molecular logic material fields.

## 3. Materials and Methods

### 3.1. Chemicals and Instruments

All reactants were commercially available and used without further purification. ^1^H and ^13^C NMR spectra (^1^H-400 MHz and ^13^C-100 MHz) were recorded on a Bruker DPX 400 MHz spectrometer in CDCl_3_ with shifts referenced to SiMe_4_ (0.00 ppm). MALDI-TOF mass spectra were recorded by a Bruker BIFLEX III ultra-high-resolution Fourier transform ion cyclotron resonance (FT-ICR) mass spectrometer using an alpha-cyano-4-hydroxycinnamic acid as the matrix. Elemental analyses were performed using an Elementar Vavio El III. Electronic absorption spectra were recorded by a U-4100 spectrophotometer. Steady-state fluorescence spectroscopic studies were performed by a F4600 (Hitachi) with a slit width of 5 nm and a photon multiplier voltage of 700 V for emission. The relative fluorescence quantum yields for ABJ-MS and its metal complexes were obtained by comparing the area under the corrected emission spectrum of the test sample with the solution of [N,N-Bis(salicylidene)-1,2-phenylenediamine]zinc(II) in DMSO with an excitation wavelength of 400 nm, which has a quantum efficiency of 0.008, according to the literature [39].

The stock DMSO/H_2_O (4:1) solution (1 mM) with either Cu^2+^, Fe^3+^, Hg^2+^, Co^2+^, Mn^2+^, Ni^2+^, Cd^2+^, Ca^2+^, Ba^2+^, Mg^2+^, Li^+^, Na^+^, K^+^, or Zn^2+^ was prepared from their chloride salts, while Pb^2+^ and Ag^+^ were prepared from their nitrate salt, and Al^3+^ from the sulfate salt. For the anion recognition test, stock solutions were prepared in DMSO–H_2_O with different anions, NaF, NaCl, NaBr, NaI, NaHSO_3_, Na_2_S, NaOAc, Na_2_SO_3_, Na_2_SO_4_, Na_2_CO_3_, Na_2_HPO_4_, or NaH_2_PO_4_. Meanwhile, ABJ-MS (2 mM) was prepared in DMSO/H_2_O (4:1). Testing solution was prepared by placing 0.05 mL of the ABJ-MS stock solutions into volumetric flasks (5 mL), adding a certain amount of tested metal stock, and a constant volume of DMSO/H_2_O (4:1), to obtain the required concentration. After mixing for thirty minutes at room temperature, the optical measurements were in progress on the spectrophotometers with a 1 cm standard quartz cell.

### 3.2. Preparation of Mono-Schiff Probe Consisting of o-Aminobenzene-Hydroxyjulolidine (ABJ-MS)

The compound of *o*-diaminobenzene (54 mg, 0.5 mmol) was dissolved in anhydrous ethanol (15 mL) before adding 9-formyl-8-hydroxyjulolidine (109 mg, 0.5 mmol), and glacial acetic acid (200 μL). The reacting mixture was heated to reflux under N_2_ protection for 5 h before being cooled to room temperature. The orange-yellow suspended substance was filtrated and washed three times in ethanol before the pure ABJ-MS samples were obtained as a yellow solid powder (95 mg, 62%). ^1^H NMR (CDCl_3_, 400 MHz) δ 13.51 (s, 1H), δ 8.33 (s, 1H), δ 7.25 (s, 1H), δ 7.02 (m, 2H, J = 24 Hz), δ 6.78 (t, 1, J = 16 Hz,), δ 3.96 (s, 2H), δ 3.24 (d, 4H, J = 3.6 Hz), δ 2.78 (m, 4H, J = 31.6 Hz), δ 1.98 (t, 4H, J = 17.6 Hz); ^13^C NMR (100 MHz, CDCl_3_) δ161.0, 158.2, 140.7, 140.5, 136.6, 129.8, 126.4, 118.8, 118.1, 115.4, 112.9, 108.9, 106.4, 50.1, 49.8, 27.2, 22.0, 21.1, 20.3; mass spectra (MALDI-TOF): an isotopic cluster peaking at *m*/*z* 307.36, (calcd. for (MH)^+^: 307.17); anal. calcd for C_19_H_21_N_3_O: C: 74.24; H: 6.89; N: 13.67. Found: C: 74.42; H: 6.59; N: 13.71.

## 4. Conclusions

In summary, a three-in-receptor mono-Schiff probe (ABJ-MS) was developed using a one-step reaction. Upon the addition of Zn^2+^/Cu^2+^/Al^3+^/Fe^3+^/Ag^+^ into the ABJ-MS solution, five distinct fluorescent behaviors with four emission wavelengths were observed simultaneity with differentiable absorption responses due to different binding affinities between these metal ions and ABJ-MS. Furthermore, a series of versatile AND/OR/IN-H-consisting logic circuits could be mimicked for ABJ-MS on the basis of diverse Cu^2+^/Zn^2+^/Al^3+^/Fe^3+^/Ag^+^-mediated optical changes. These show that the smart ABJ-MS molecules are not only quite versatile in detecting Cu^2+^/Zn^2+^/Al^3+^/Fe^3+^/Ag^+^ in nature but also offer great potential in molecular logic fields.

## Figures and Tables

**Table 1:** Detection by ABJ-MS of Zn^2+^/Al^3+^/Fe^3+^ in river water samples.

Sample	Zn^2+^ added (μM)	Zn^2+^ found(μM)	Recovery (%)	R.S.D. ^a^ (%)
River water	0.00	0.00		
	10.00	10.06	101	0.6
Artificial water ^b^	0.00	10.05	101	0.5
	10.00	20.28	102	1.4
Sample	Al^3+^ added (μM)	Al^3+^ found (μM)	Recovery (%)	R.S.D. ^a^ (%)
River water	0.00	0.00		
	10.00	10.08	101	0.8
Artificial water ^c^	0.00	10.05	101	0.5
	10.00	20.30	102	1.5
Sample	Fe^3+^ added (μM)	Fe^3+^ found (μM)	Recovery (%)	R.S.D. ^a^ (%)
River water	0.00	0.00		
	10.00	10.08	101	0.8
Artificial water ^d^	0.00	10.05	101	0.5
	10.00	20.30	102	1.5

^a^ n = 3; R.S.D.: relative stand deviation; ^b–d^ synthesized by the addition of Zn^2+^/Al^3+^/Fe^3+^ as well as Hg^2+^, Zn^2+^, Ni^2+^, Pb^2+^, Cd^2+^, Ba^2+^, Mg^2+^, Ca^2+^, Li^+^, Na^+^, and K^+^ (10.00 μM) into river water; conditions: 20.00 μM ABJ-MS in mixed solution of DMSO/river water (4:1).

## Data Availability

Not applicable.

## Supplementary Materials

The following supporting information can be downloaded at: https://www.mdpi.com/article/10.3390/molecules28176400/s1, Figure S1: ^1^H and ^12^C NMR spectra of ABJ-MS in CDCl_3_ in addition of mass spectroscopy; Figure S2: The mass spectroscopy of ABJ-MS; Figure S3: The optical properties of ABJ-MS in DMSO/H_2_O mixture; Figure S4: The absorption titration spectra of ABJ-MS with Zn^2+^; Figure S5: The mass spectroscopy of ABJ-MS–Zn^2+^ system; Figure S6: The absorption titration properties of ABJ-MS with Al^3+^; Figure S7: The absorption spectra of ABJ-MS–Fe^3+^/Zn^2+^/Al^3+^/Ag^+^ system upon addition of Cu^2+^; Figure S8: The absorption spectra of ABJ-MS–Al^3+^/Ag^+^ upon addition of Zn^2+^; Figure S9: The absorption spectra of ABJ-MS–Ag^+^ system upon addition of Al^3+^; Figures S10–S14: The fluorescence emission spectra of ABJ-MS–Zn^2+^/Cu^2+^/Fe^3+^/Al^3+^/Ag^+^ upon addition of different metal ions; Figure S15: The optical properties of ABJ-MS upon adding anions; Figure S16: The absorption and fluorescence emission spectra of ABJ-MS upon addition of HCl; Figure S17: Schematic diagram of the IN-H and OR logic gate for ABJ-MS with Zn^2+^ and Al^3+^; Table S1: Truth table for ABJ-MS with Ag^+^, Fe^3+^, Al^3+^, and Zn^2+^ four inputs, and the fluorescence intensity at 3000/1500 as the threshold to define the “1” (˃3000) and “0” (˂3000) state; Table S2: Truth table for ABJ-MS with Ag^+^, Fe^3+^, Al^3+^, and Zn^2+^ four inputs, and the fluorescence intensity at 1500 as the threshold to define the “1” (˃1500) and “0” (˂1500) states.

## Sample Availability

Samples of the compound ABJ-MS are available from the authors.
