# Supplementary material for: A Simple Schiff Base Probe for Quintuplicate-Metal Analytes with Four Emission-Wavelength Responses
Source: Molecules. 2023 Sep 1;28(17):6400. doi: 10.3390/molecules28176400 (PMC10490265; doi:10.3390/molecules28176400)
Supplement: Supplementary file 1 [file molecules-28-06400-s001.zip › molecules-2567232-supplementary.pdf]

Supporting Information

A Simple Schiff Base Probe for Quintuplicate-Metal Analytes with Four Emission-Wavelength Responses

Jingzhe Zhang,[1,2,3] Kaili Wang,[2,4]* Yilu Sun[1,3]*

[1] Key Laboratory of Environmental Biotechnology, Research Center for Eco-Environmental Sciences, Chinese Academy of Sciences, Beijing 100085, China.

[2] Beijing Municipal Research Institute of Eco-Environmental Protection, Beijing 100037, China.

[3] University of Chinese Academy of Sciences, Beijing, 100049, China.

[4] State Environmental Protection Engineering (Beijing) Center for Industrial Wastewater Pollution Control， Beijing 100037, China

Corresponding Authors: * E-mail: wangkaili@cee.cn (K.W);
* E-mail: ylsun@rcees.ac.cn (Y.S).

Content

1. The ^{1}H and ^{13}C NMR spectra of ABJ-MS, Figure S1, Page S3.
2. The mass spectroscopy of ABJ-MS, Figure S2, Page S4.
3. The optical properties of ABJ-MS in DMSO/H_2O mixture, Figure S3, Page S5.
4. The absorption titration spectra of ABJ-MS upon increasing amount of Zn^{2+}, Figure S4, Page S6.
5. The mass spectroscopy of ABJ-MS-Zn^{2+} system, Figure S5, Page S7.
6. The absorption titration spectra of ABJ-MS upon increasing amount of Al^{3+}, Figure S6, Page S8.
7. The absorption spectra of ABJ-MS-Fe^{3+}/Zn^{2+}/Al^{3+}/Ag^{+} upon addition of Cu^{2+}/Zn^{2+}/Al^{3+}, Figures S7-9, Pages S9-11.
8. The fluorescence spectra of ABJ-MS-Zn^{2+}/Cu^{2+}/Fe^{3+}/Al^{3+}/Ag^{+} upon addition of the other tested metal ions, Figures S10-14, Pages S12-16.
9. The optical properties of ABJ-MS upon adding anions, Figure S15, Page S17.
10. The absorption and fluorescence emission spectra of ABJ-MS (20 μM) upon addition of HCl in mixed DMSO/H_2O (4:1), Figure S16, Pages S18.
11. Schematic diagram of the INH and OR logic gates with two inputs and two maximum fluorescence intensity outputs, Figure S17, Page S19.
12. Truth table for ABJ-MS with Ag^{+}, Fe^{3+}, Al^{3+}, and Zn^{2+} four inputs, the maximum fluorescence intensity as outputs, and the threshold of the fluorescence intensity being 3000 and 1500, respectively, Tables S1-S2, Pages S20-21.

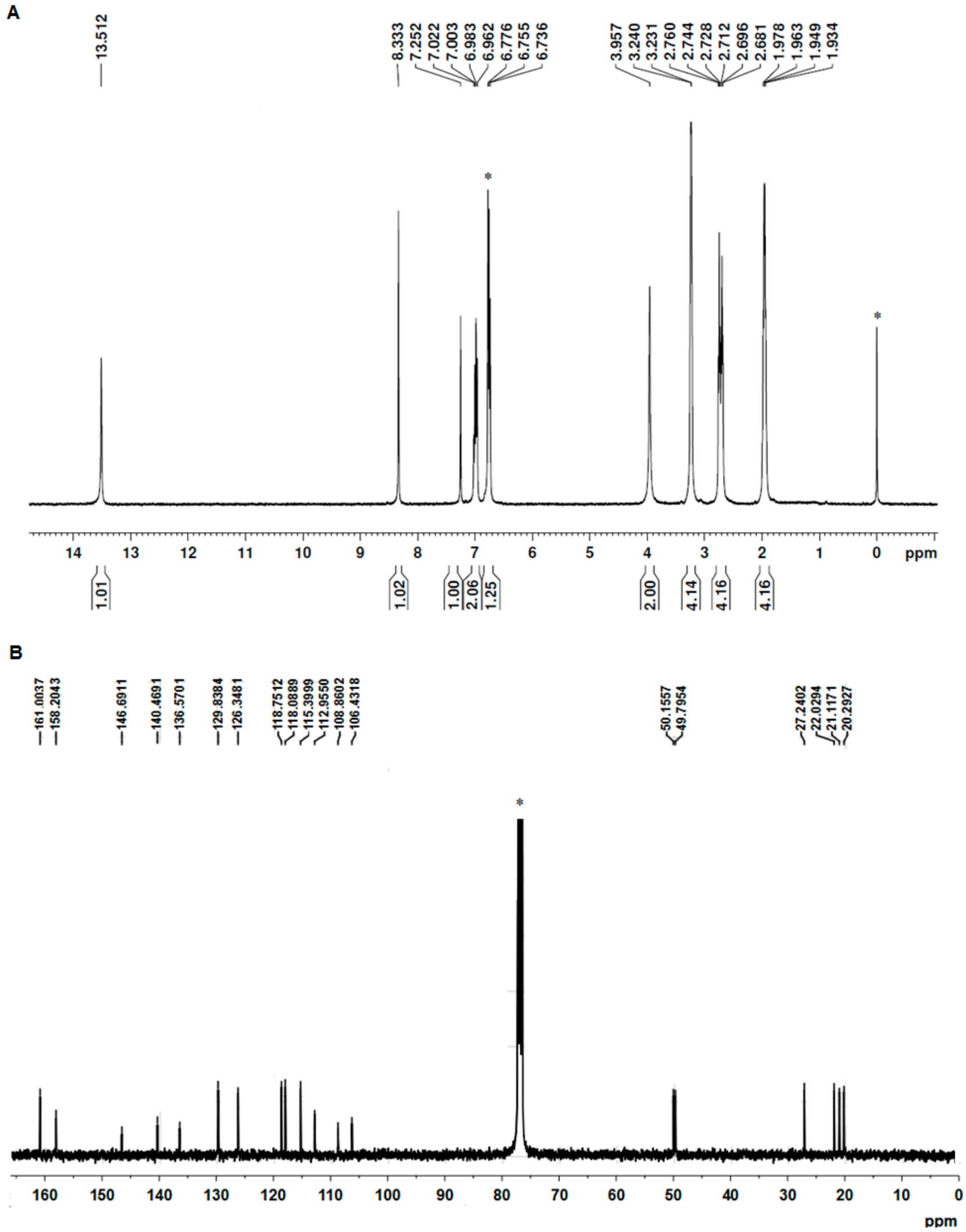

Figure S1 ^{1}H NMR (A) and ^{13}C NMR spectra (B) of ABJ-MS in $CDCl_3$. * indicates the residual solvent signals.

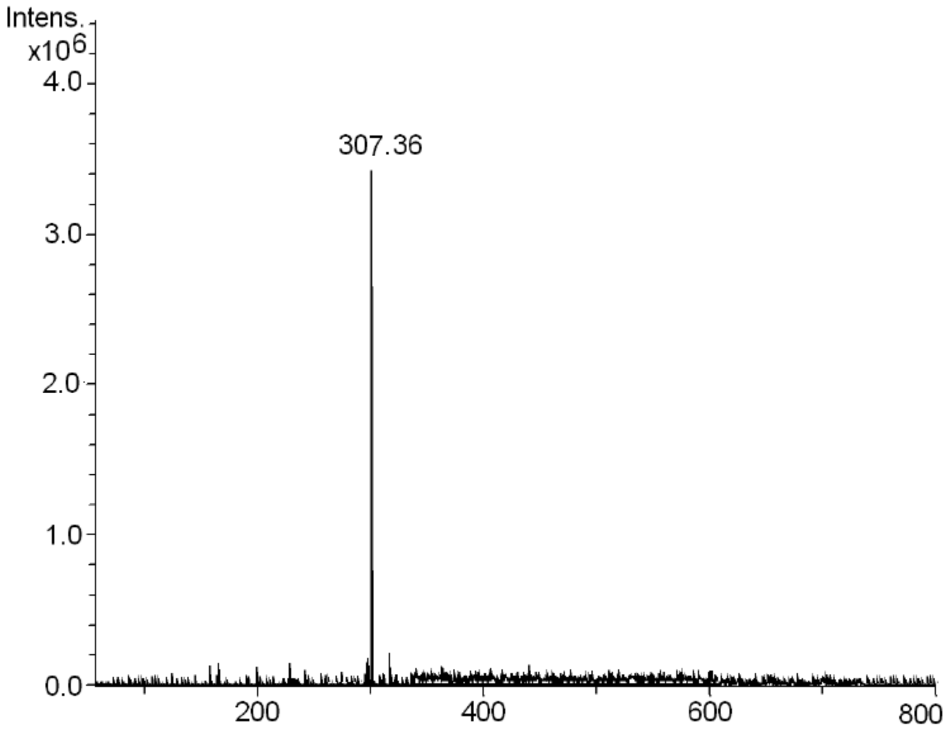

Figure S2 Mass spectroscopy of ABJ-MS.

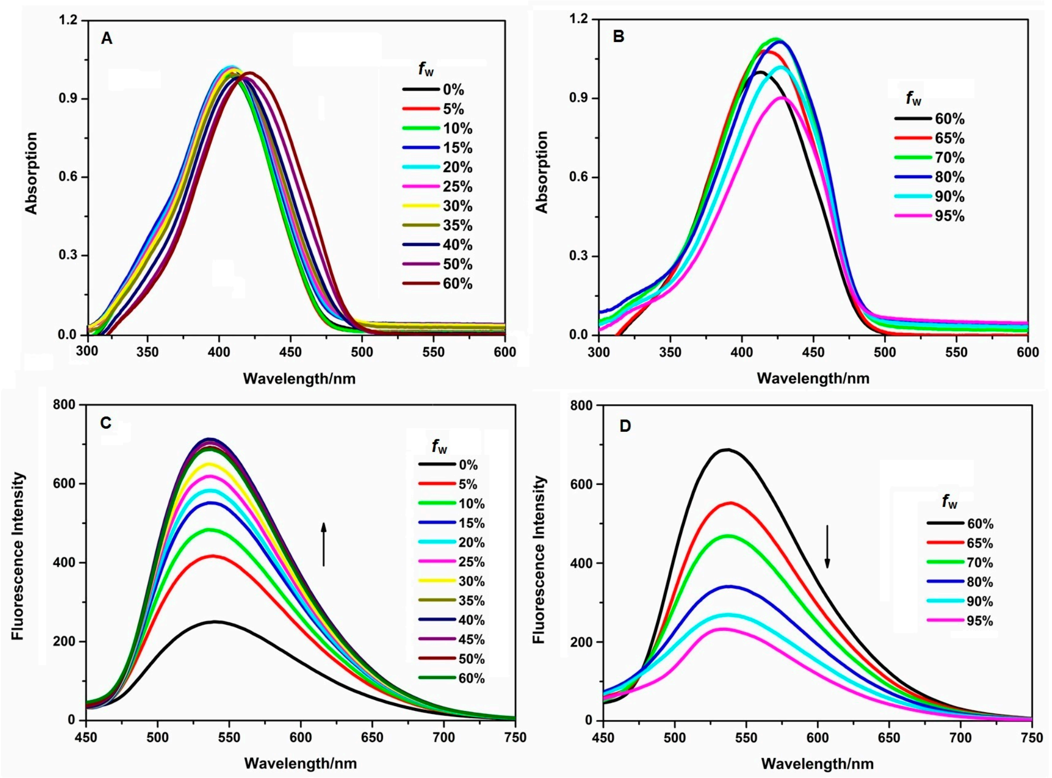

Figure S3 The electronic absorption (A,B) and fluorescence emission (C,D) of ABJ-MS (20 μM) in DMSO-H_2O mixture with H_2O fractions (f_w) changing from 0% to 95%.

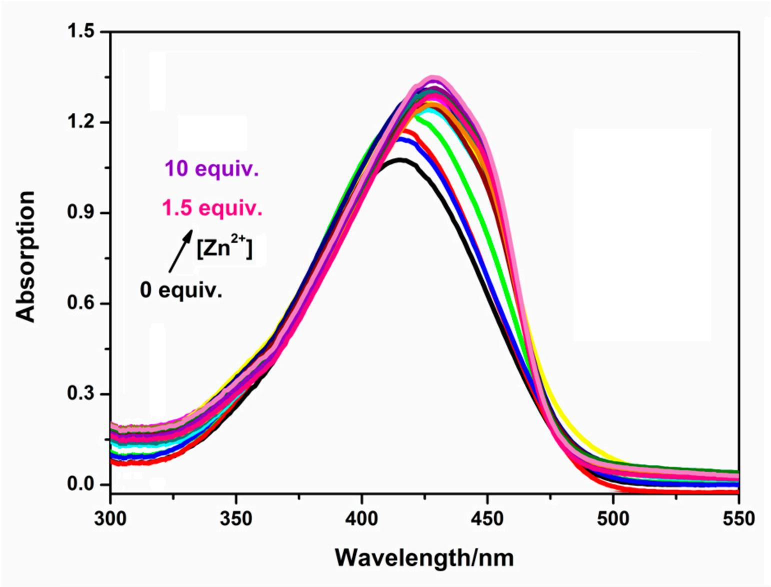

Figure S4 The absorption spectrum of ABJ-MS (20 μM) along with increasing Zn^{2+} amount in mixed DMSO/H_2O (4:1).

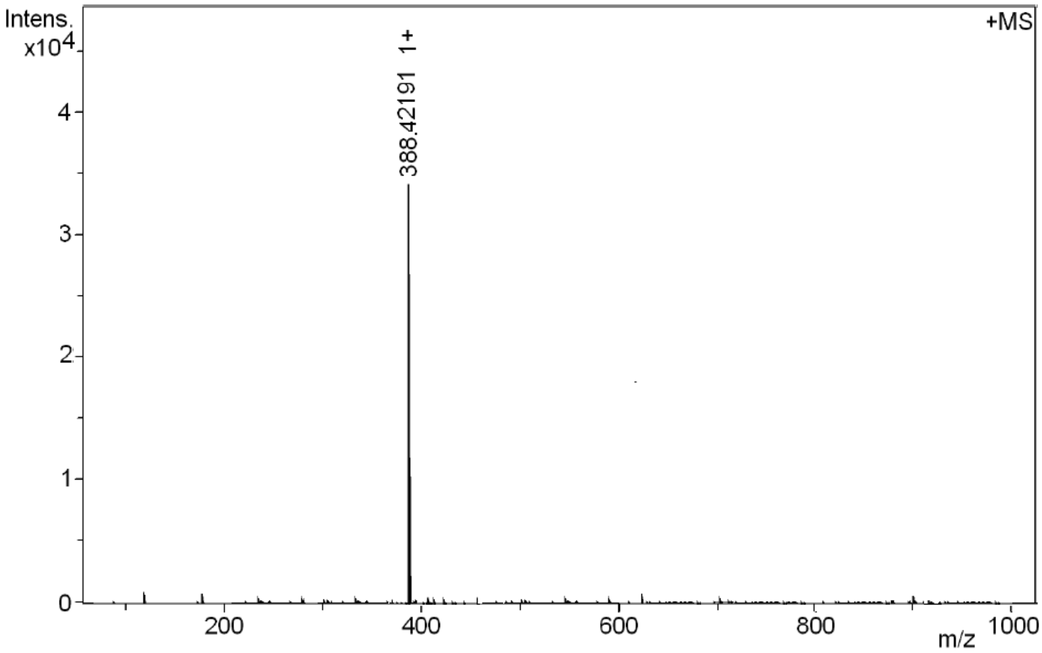

Figure S5 Mass spectroscopy of ABJ-MS-Zn^{2+} system.

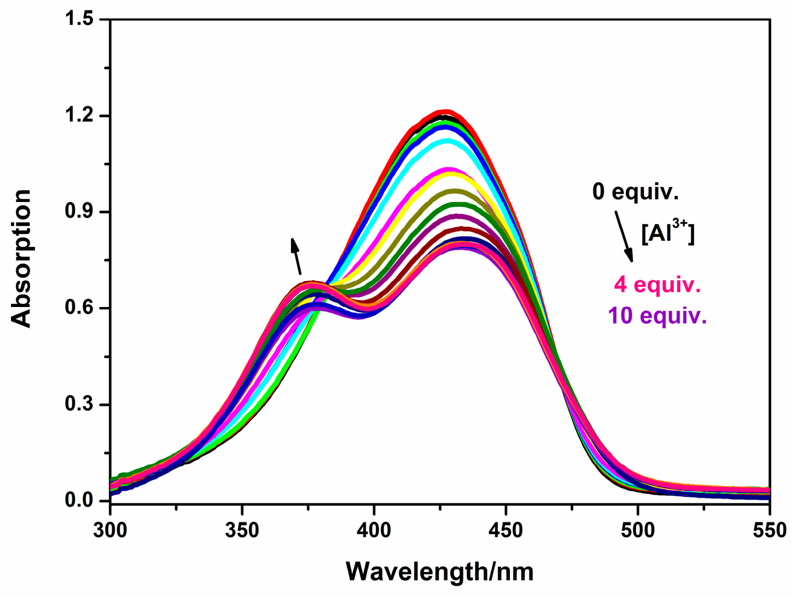

Figure S6 The absorption spectrum of ABJ-MS (20 μM) along with increasing Al^{3+} amount in mixed DMSO/H_2O (4:1).

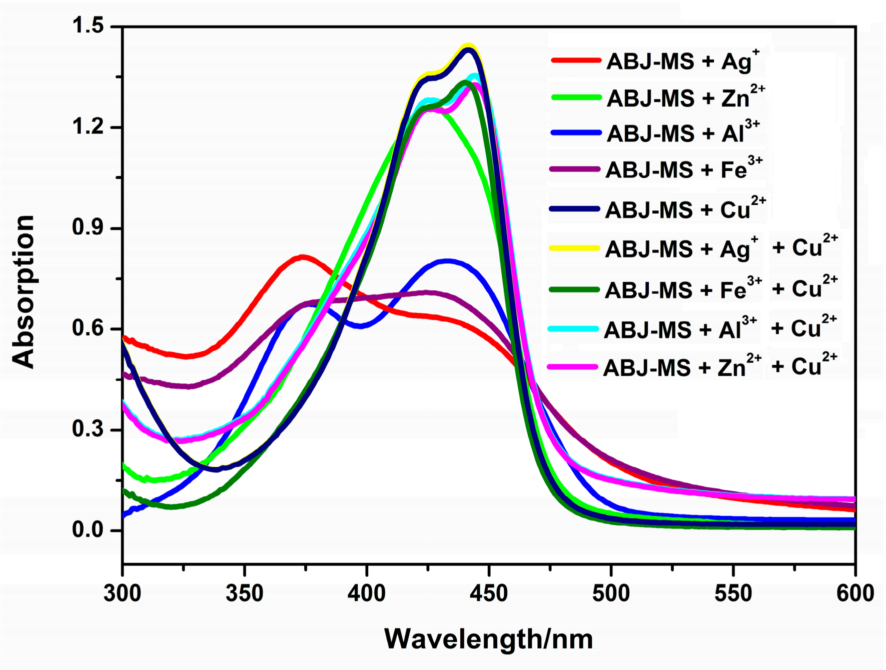

Figure S7 The absorption spectra of ABJ-MS-Fe^{3+}/Zn^{2+}/Al^{3+}/Ag^{+} system upon addition of Cu^{2+} in mixed DMSO/H_2O (4:1).

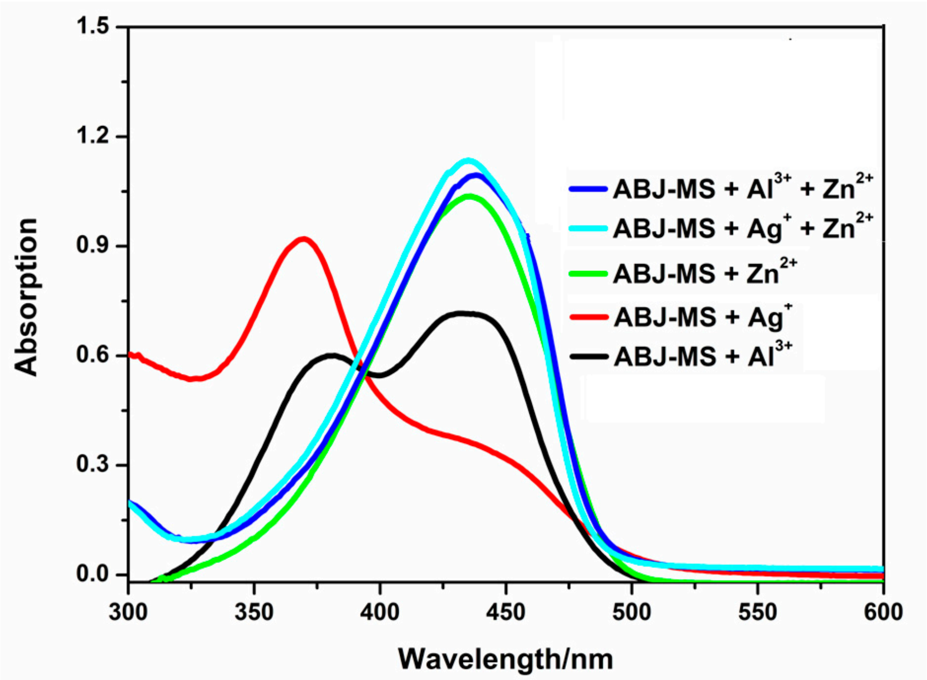

Figure S8 The absorption spectra of ABJ-MS-Al^{3+}/Ag^{+} upon addition of Zn^{2+} in mixed DMSO/H_2O (4:1).

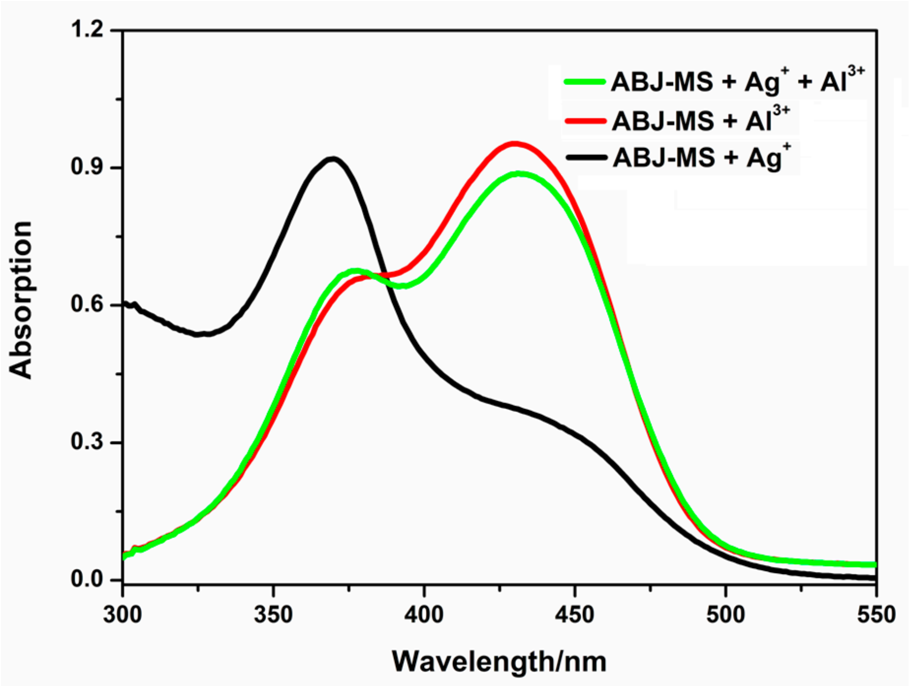

Figure S9 The absorption spectra of ABJ-MS-Ag^{+} system upon addition of Al^{3+} in mixed DMSO/H_2O (4:1).

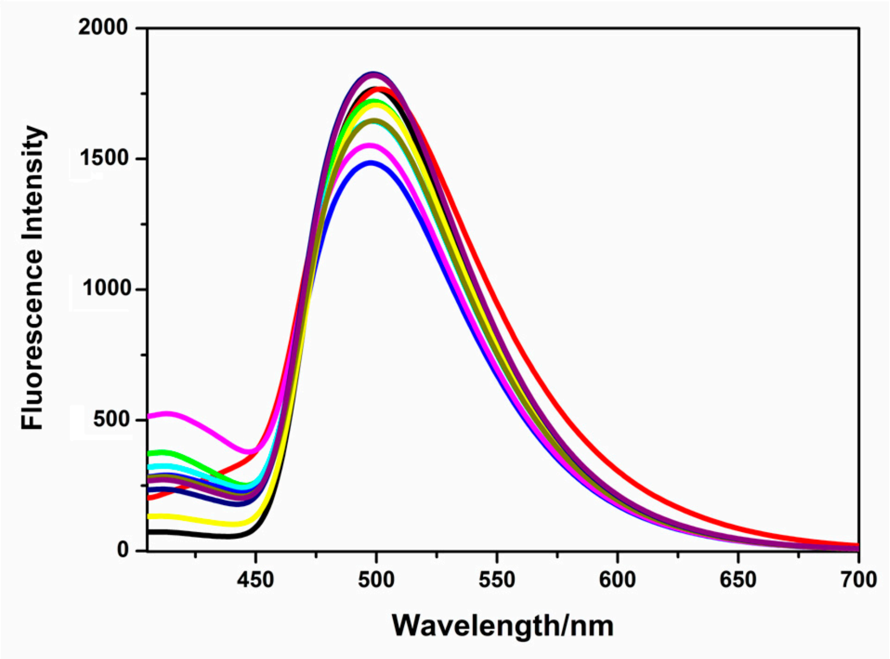

Figure S10 The fluorescence emission spectra of ABJ-MS-Zn^{2+} (1:10) system in mixed DMSO/H_2O (4:1) upon addition of other tested metal ions such as Hg^{2+}, Pb^{2+}, Co^{2+}, Mn^{2+}, Ni^{2+}, Cd^{2+}, Ca^{2+}, Ba^{2+}, Mg^{2+}, Li^{+}, Na^{+} or K^{+} (10 equiv.), respectively.

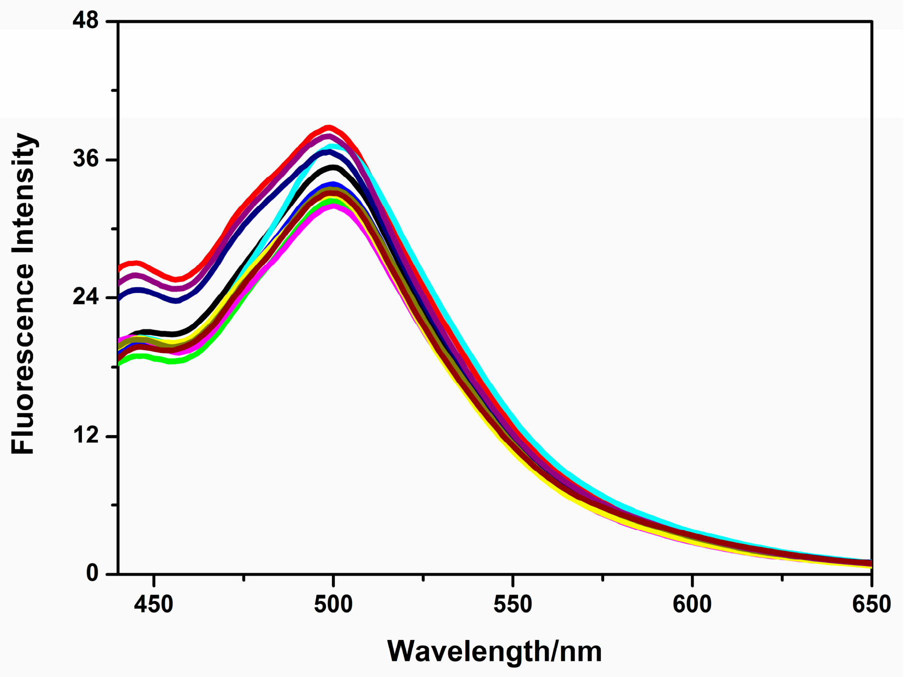

Figure S11 The fluorescence emission spectra of ABJ-MS-Cu^{2+} (1:10) system in mixed DMSO/H_2O (4:1) upon addition of other tested metal ions such as Hg^{2+}, Pb^{2+}, Co^{2+}, Mn^{2+}, Ni^{2+}, Cd^{2+}, Ca^{2+}, Ba^{2+}, Mg^{2+}, Li^{+}, Na^{+} or K^{+} (10 equiv.), respectively.

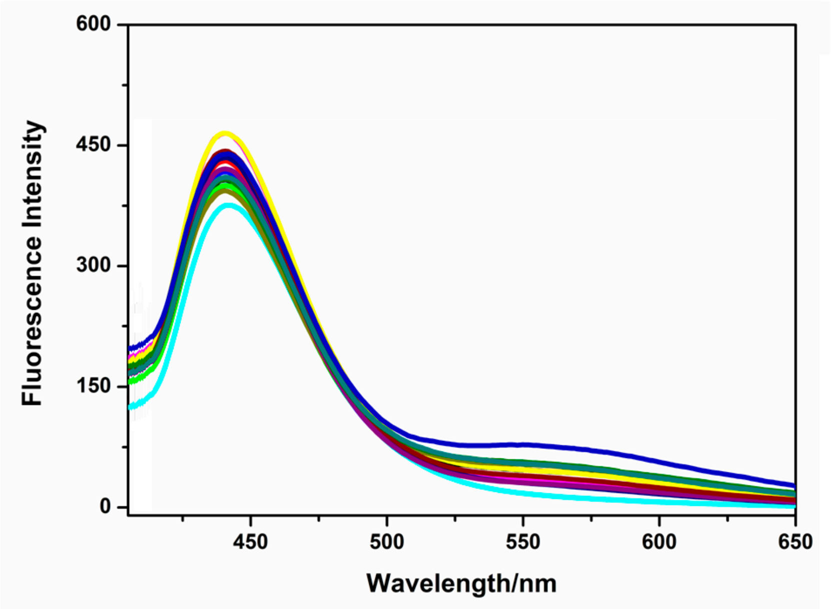

Figure S12 The fluorescence emission of ABJ-MS-Fe^{3+} (1:10) system in mixed DMSO/H_2O (4:1) upon addition of other tested metal ions such as Hg^{2+}, Pb^{2+}, Co^{2+}, Mn^{2+}, Ni^{2+}, Cd^{2+}, Ca^{2+}, Ba^{2+}, Mg^{2+}, Li^{+}, Na^{+} or K^{+} (10 equiv.), respectively.

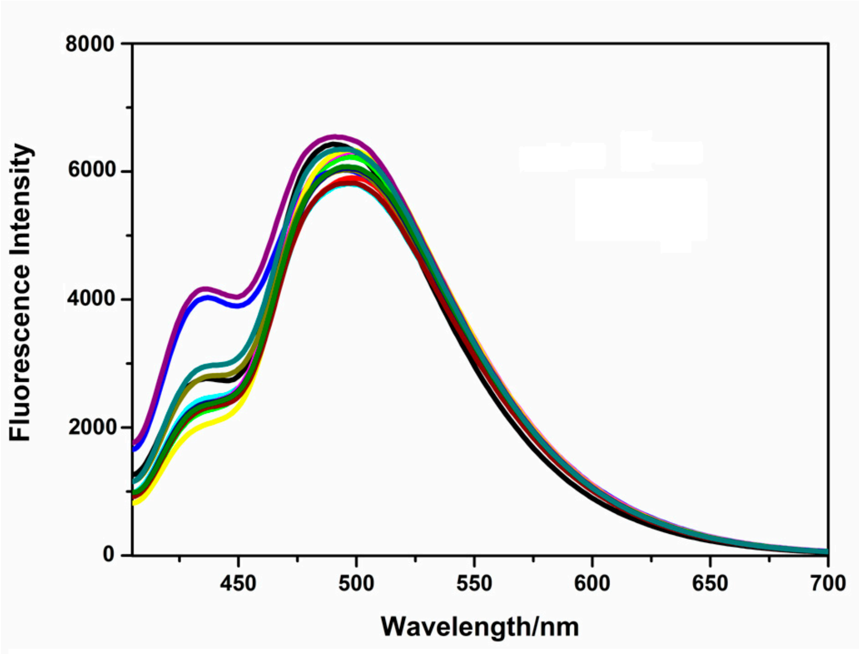

Figure S13 The fluorescence emission of ABJ-MS-Al^{3+} (1:10) system in mixed DMSO/H_2O (4:1) upon addition of other tested metal ions such as Hg^{2+}, Pb^{2+}, Co^{2+}, Mn^{2+}, Ni^{2+}, Cd^{2+}, Ca^{2+}, Ba^{2+}, Mg^{2+}, Li^{+}, Na^{+} or K^{+} (10 equiv.), respectively.

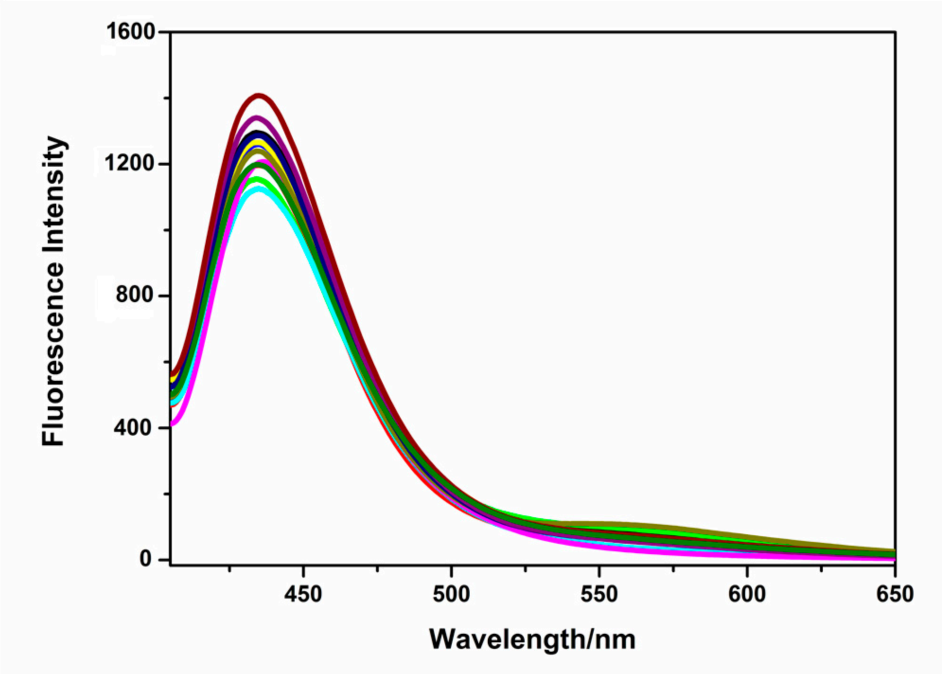

Figure S14 The fluorescence emission of ABJ-MS-Ag^{+} (1:10) system in mixed DMSO/H_2O (4:1) upon addition of other tested metal ions such as Hg^{2+}, Pb^{2+}, Co^{2+}, Mn^{2+}, Ni^{2+}, Cd^{2+}, Ca^{2+}, Ba^{2+}, Mg^{2+}, Li^{+}, Na^{+} or K^{+} (10 equiv.), respectively.

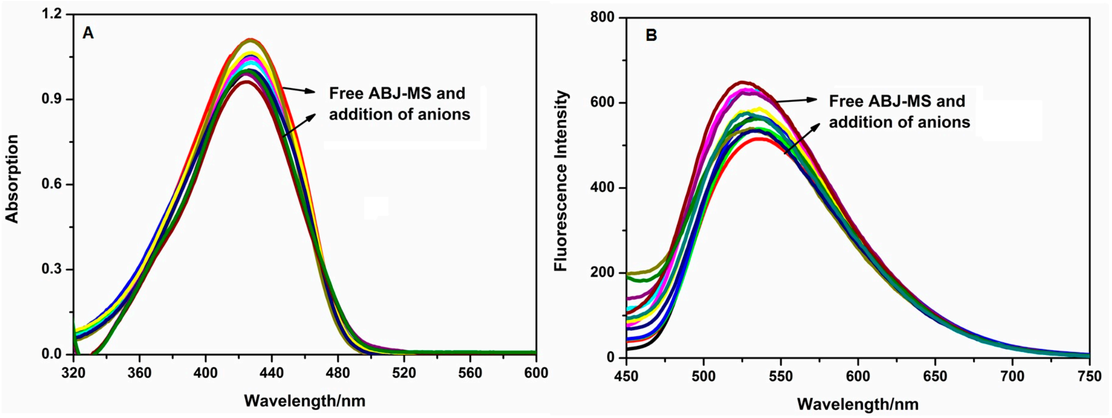

Figure S15 The electronic absorption and fluorescence emission of ABJ-MS upon addition of different anions such as F^-, Cl^-, Br^-, I^-, HSO_3^-, OAc^-, S^{-2}, SO_3^{-2}, SO_4^{-2}, CO_3^{-2}, HPO_4^{-2}, $H_2PO_4^-$ in mixed DMSO/H_2O (4:1).

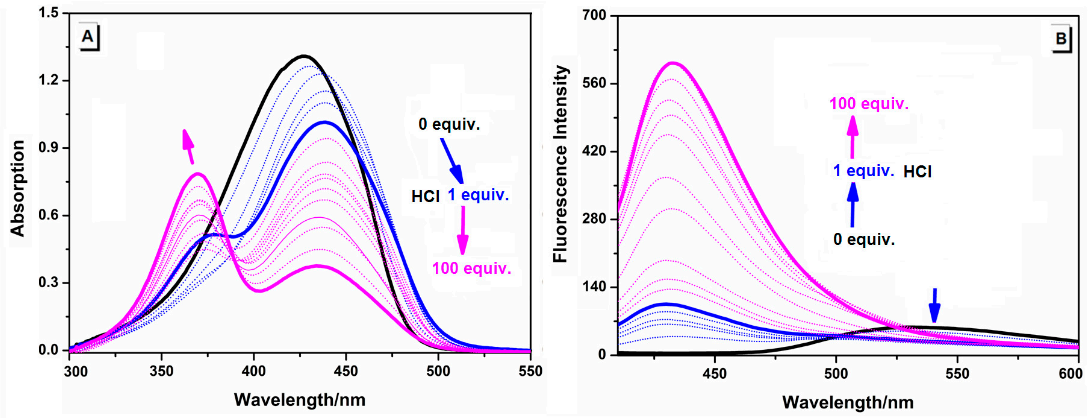

Figure S16 The absorption and fluorescence emission spectra of ABJ-MS (20 μM) upon addition of HCl in mixed DMSO/H_2O (4:1).

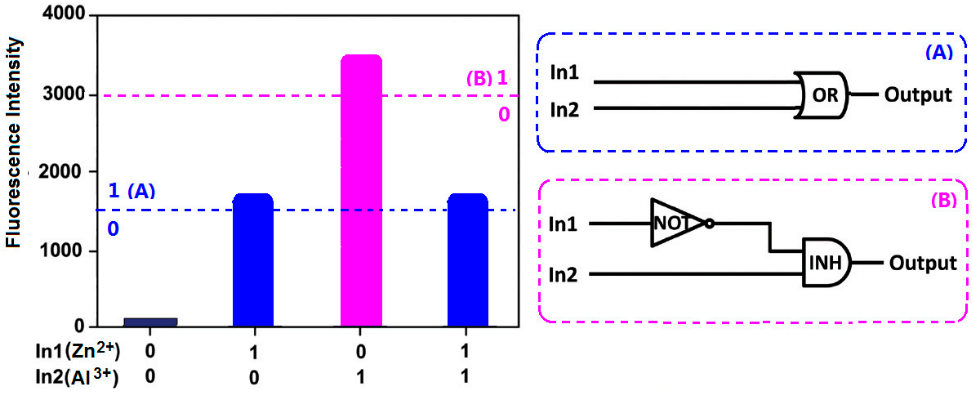

Figure S17 Schematic diagram of the INH and OR logic gate for ABJ-MS (20 μM) with 10 equiv. of Zn^{2+} and Al^{3+} as inputs (In1 and In2), the maximum fluorescence emission intensity of ABJ-MS-M system as outputs, and the threshold level of fluorescence intensity being 1500 (A) and 3000 (B), respectively. Dotted lines represent the threshold level of fluorescence outputs.

Table S1 Truth table for ABJ-MS[a] with Ag^+, Fe^{3+}, Al^{3+} and Zn^{2+} four inputs (In1, In2, In3 and In4), the maximum fluorescence emission outputs, and the fluorescence intensity at 3000 as the threshold to define the "1" (> 3000) and "0" (< 3000) state.

Inputs				outputs			
Fe^{3+b}	Zn^{2+b}	Al^{3+b}	Ag^{+b}	$F_{500}{}^{c}$	$F_{490}{}^{c}$	$F_{440}{}^{c}$	$F_{430}{}^{c}$
0	0	0	0	0	0	0	0
1	0	0	0	0	0	0	0
0	1	0	0	0	0	0	0
0	0	1	0	0	1	0	0
0	0	0	1	0	0	0	0
1	1	0	0	0	0	0	0
1	0	1	0	0	0	0	0
1	0	0	1	0	0	0	0
0	1	1	0	0	0	0	0
0	1	0	1	0	0	0	0
0	0	1	1	0	1	0	0
1	1	1	0	0	0	0	0
1	0	1	1	0	0	0	0
1	1	0	1	0	0	0	0
0	1	1	1	0	0	0	0
1	1	1	1	0	0	0	0

[a] The concentration of ABJ-MS being 20 μM in DMSO/H_2O (4:1) solution; [b] The concentration of different metal ions being 200 μM; [c]The maximum fluorescence emission wavelength of ABJ-MS upon addition of metal ions (nm).

Table S2 Truth table for ABJ-MS[a] with Ag^+, Fe^{3+}, Al^{3+} and Zn^{2+} four inputs (In1, In2, In3 and In4), the maximum fluorescence emission outputs, and the fluorescence intensity at 1500 as the threshold to define the "1" (> 1500) and "0" (< 1500) state.

Inputs				outputs			
Fe^{3+b}	Zn^{2+b}	Al^{3+b}	Ag^{+b}	$F_{500}{}^{c}$	$F_{490}{}^{c}$	$F_{440}{}^{c}$	$F_{430}{}^{c}$
0	0	0	0	0	0	0	0
1	0	0	0	0	0	0	0
0	1	0	0	1	0	0	0
0	0	1	0	0	1	0	0
0	0	0	1	0	0	0	0
1	1	0	0	0	0	0	0
1	0	1	0	0	0	0	0
1	0	0	1	0	0	0	0
0	1	1	0	1	0	0	0
0	1	0	1	1	0	0	0
0	0	1	1	0	1	0	0
1	1	1	0	0	0	0	0
1	0	1	1	0	0	0	0
1	1	0	1	0	0	0	0
0	1	1	1	1	0	0	0
1	1	1	1	0	0	0	0

[a] The concentration of ABJ-MS being 20 μM in DMSO/H_2O (4:1) solution; [b] The concentration of different metal ions being 200 μM; [c] The maximum fluorescence emission wavelength of ABJ-MS upon addition of metal ions (nm).
